# Complete plastome sequence of *Psidium guajava* L. (Myrtaceae)

**DOI:** 10.1080/23802359.2016.1209096

**Published:** 2016-09-05

**Authors:** Sangjin Jo, Hoe-Won Kim, Young-Kee Kim, Se-Hwan Cheon, Ki-Joong Kim

**Affiliations:** School of Life Sciences, Korea University, Seoul, Korea

**Keywords:** Guava, Myrtaceae, plastome; *Psidium guajava;* tropical fruit

## Abstract

In this study, we determined the complete plastome sequence of *Psidium guajava* L. (Myrtaceae) (NCBI acc. no. KX364403). The gene order and structure of the *P. guajava* plastome are similar to those of a typical angiosperm. The complete plastome is 158,841 bp in length, and consists of a large single copy of 87,675 bp and a small single copy of 18,464 bp, separated by two inverted repeats of 26,351 bp. The overall AT content of the sequence is 63.0%. The plastome contains 112 genes, of which 78 are protein-coding genes, 30 are tRNA genes, and four are rRNA genes. Sixteen genes contain one intron and two genes have two introns. A total of 100 simple sequence loci were identified from the genome. Phylogenetic analysis revealed that *P. guajava* is a sister group of *Eugenia uniflora* with 100% bootstrap support.

Guava (*Psidium guajava* L.), one of the popular tropical fruits in the family Myrtaceae, is a native of Central America and Northern South America (Kim 2011). Guava has been treated as one of the super fruits because of its diverse array of nutrients. It is extraordinarily rich in vitamin C, lycopene, and antioxidants that are beneficial for human health. Guavas are also rich in several minerals such as manganese, iron, and potassium. Although the plastome data for Myrtales are relatively rich compared to those of other orders, most of the determined sequences are concentrated in the genera *Eucalyptus*, *Corymbia*, and *Oenothera* (Greiner et al. 2008; Bayly et al. 2013). Only a single plastome sequence is available from the tribe Myrteae, namely that of *Eugenia uniflora.*

In terms of species number, Myrtaceae is the 8th largest family of flowering plants. The family consists of 132 genera and 5950 species (Christenhusz & Byng 2016), with most species being distributed in the subtropical and tropical regions. The family includes many plants of economic value, such as species of *Eucalyptus*, *Eugenia,* and *Psidium,* which are important sources of timbers, essential oils, and fruits. The complete plastome sequence of *P. guajava* will aid in the development of molecular markers for the identification and improvement of cultivars of this plant. In addition, the plastome data from Myrteae will be valuable in elucidating plastome evolution and phylogenetic relationships in the Myrtaceae.

The leaves of *P. guajava* used in this study were collected from the Korea University greenhouse, where we grew the plants from seeds originally collected in Thailand. The plants flowered and fruited in the greenhouse. A voucher specimen was deposited in the Korea University Herbarium (KUS acc. no. 2014-0250). Fresh leaves were ground into powder using liquid nitrogen and total DNAs were extracted using the CTAB method (Doyle & Doyle 1987). The DNAs were further purified by the ultracentrifugation and dialysis (Palmer 1986). The genomic DNAs are deposited in the Plant DNA Bank in Korea (PDBK acc. no. 2014-0250). The complete plastome sequence was generated using an Illumina HiSeq 2000 system (Illumina Inc., San Diego, CA). Average redundancy of the sequence coverage was 1414 times. Annotations were performed using the National Center for Biotechnology Information (NCBI) BLAST, DOGMA (Wyman et al. 2004), and tRNAscan-SE programs (Lowe & Eddy 1997). For the phylogenetic analysis, we selected and downloaded 32 complete plastome sequences based on the APG IV system (Byng et al. 2016) from the NCBI database.

The gene order and structure of the *P. guajava* plastome are similar to those of a typical angiosperm (Shinozaki et al. 1986; Kim & Lee 2004; Yi & Kim 2012). The complete plastome is 158,841 bp in length and consists of a large single copy (LSC) of 87,675 bp and a small single copy (SSC) of 18,464 bp, separated by two inverted repeats (IR) of 26,351 bp. The plastome comprises of 112 unique genes (78 protein-coding genes, 30 tRNA genes, and four rRNA genes). Among the protein-encoding genes, *infA* is a pseudogene. The average A–T content of the plastome is 63.0%. The A–T contents in the LSC, SSC, and IR regions are 65.1%, 69.3%, and 57.2%, respectively. An average coverage of sequence is 1414 times. A total of 16 genes contain intron and two genes, *ycf3* and *clpP*, have two introns. A total of 100 simple sequence repeat (SSR) loci, which can be defined as having more than 10 duplications of simple nucleotide(s), are scattered among the noncoding regions of the genome. Among these, 71, 4, and 25 are mono-SSR, di-SSR, and tri-SSR loci, respectively. Some of these loci will be useful in identifying cultivars of *P. guajava*.

To validate the phylogenetic relationships of *P. guajava* among malvids, we constructed a maximum likelihood (ML) tree. Phylogenetic analysis was performed on a data set that included the 78 protein-coding genes (excluding *infA*) and four rRNA genes from 33 taxa using RAxML v. 7.7.1 (Stamatakis et al. 2008). The 82 gene sequences (81,722 bp) were aligned with MUSCLE in Geneious v. 6.1.8 (Biomatters Ltd.; Kearse et al. 2012). The results showed that *P. guajava* forms a sister group relationship with *Eugenia uniflora* with 100% bootstrap support. Both species belong to the same tribe Myrteae within Myrtaceae, whereas all other taxa belong to the tribe Eucalypteae (Figure 1). The phylogenetic relationships within the Myrtaceae were established using the nuclear ITS region and plastid *matK* and *ndhF* genes (Wilson et al. 2005; Biffin et al. 2010). Two subfamilies and 17 tribes were recognized within the Myrtaceae. Several relationships at the tribal level, nevertheless, remain unresolved. The complete plastome sequences will be helpful in resolving these relationships; however, to date, complete plastome data are available only from two of the 17 tribes. Therefore, more plastome sequences from diverse tribes of Myrtaceae will prove valuable in resolving the outstanding phylogenetic questions in this family.

**Figure 1.**
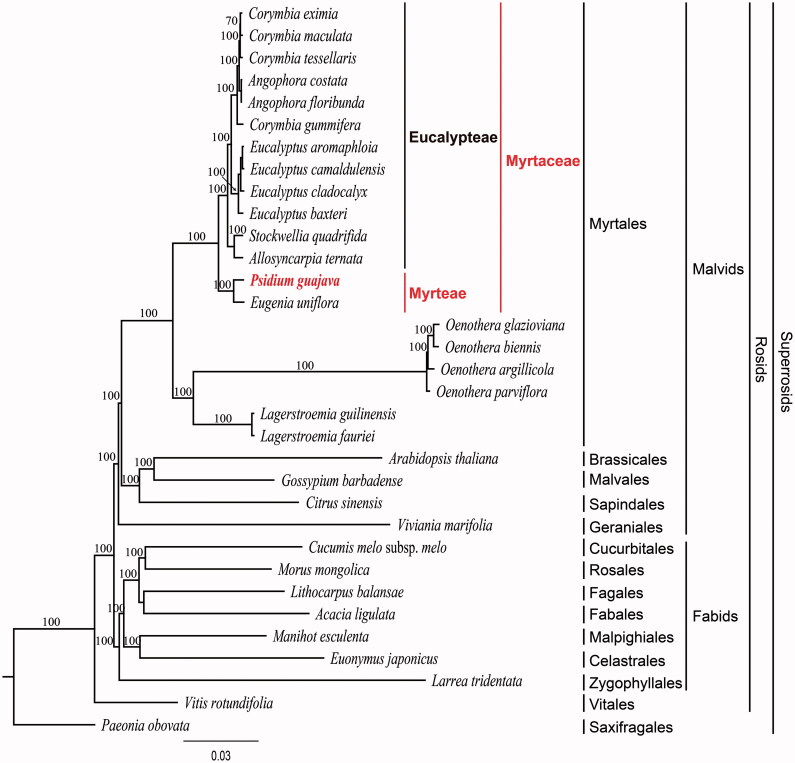
Maximum Likelihood (ML) tree based on 78 protein-coding and four rRNA genes from 33 plstomes as determined by RAxML. The numbers at each node indicate the ML bootstrap values. Genbank accession numbers of taxa are shown below, *Acacia ligulata* (NC_026134), *Allosyncarpia ternata* (NC_022413), *Angophora costata* (NC_022412), *Angophora floribunda* (NC_022411), *Arabidopsis thaliana* (NC_000932), *Citrus sinensis* (NC_008334), *Corymbia eximia* (NC_022409), *Corymbia gummifera* (NC_022407), *Corymbia maculata* (NC_022408), *Corymbia tessellaris* (NC_022410), *Cucumis melo* subsp. *melo* (NC_015983), *Eucalyptus aromaphloia* (NC_022396), *Eucalyptus baxteri* (NC_022382), *Eucalyptus camaldulensis* (NC_022398), *Eucalyptus cladocalyx* (NC_022394), *Eugenia uniflora* (NC_027744), *Euonymus japonicus* (NC_028067), *Gossypium barbadense* (NC_008641), *Lagerstroemia fauriei* (NC_029808), *Lagerstroemia guilinensis* (NC_029885), *Larrea tridentata* (NC_028023), *Lithocarpus balansae* (NC_026577), *Manihot esculenta* (NC_010433), *Morus mongolica* (NC_025772), *Oenothera argillicola* (NC_010358), *Oenothera biennis* (NC_010361), *Oenothera glazioviana* (NC_010360), *Oenothera parviflora* (NC_010362), *Paeonia obovata* (NC_026076), *Psidium guajava* (KX364403), *Stockwellia quadrifida* (NC_022414), *Vitis rotundifolia* (NC_023790) and *Viviania marifolia* (NC_023259).
